# Baseline and Early On-Treatment Prognostic Nutritional Index in Hormone Receptor–Positive, HER2-Negative Metastatic Breast Cancer Treated with CDK4/6 Inhibitors

**DOI:** 10.3390/jcm15176790

**Published:** 2026-09-01

**Authors:** Ezgi Çoban, Atilla Eren Kurt, Fırat Akagündüz, Ahmet Demirel, Mustafa Alperen Tunç, Burak Paçacı, Ali Kaan Güren, Erkam Kocaaslan, Pınar Erel, Yeşim Ağyol, Abdussamet Çelebi, Selver Işık, Nazım Can Demircan, Osman Köstek, İbrahim Vedat Bayoğlu, Murat Sarı

**Affiliations:** 1 Division of Medical Oncology, Department of Internal Medicine, Marmara University School of Medicine, Istanbul 34854, Türkiyem.alperen.tunc@gmail.com (M.A.T.); drpacaci@gmail.com (B.P.); dr-selver83@hotmail.com (S.I.);; 2 Department of Internal Medicine, Marmara University School of Medicine, Istanbul 34854, Türkiye; 3 Department of Internal Medicine, Istanbul Bağcılar Training and Research Hospital, Istanbul 34200, Türkiye; 4 Department of Medical Oncology, Kars Harakani State Hospital, Kars 36100, Türkiye

**Keywords:** prognostic nutritional index, breast cancer, CDK4/6 inhibitor, progression-free survival, overall survival, systemic inflammation, nutritional status

## Abstract

**Background/Objectives:** The prognostic nutritional index (PNI) has been associated with outcome in metastatic breast cancer, but studies in CDK4/6 inhibitor-treated patients have used cohort-derived thresholds and examined only the pretreatment value. We assessed the PNI as a continuous variable, compared it with inflammatory indices, and examined whether repeated measurement added information. **Methods:** We reviewed 192 consecutive patients with hormone receptor–positive, HER2-negative metastatic breast cancer treated with a CDK4/6 inhibitor and endocrine therapy at a single centre. We calculated the PNI, neutrophil-to-lymphocyte ratio (NLR), and systemic inflammation response index (SIRI) before treatment and recalculated them before cycles 2 and 3. The Cox models were adjusted for age, liver metastasis and line of therapy; no threshold was derived from these data. **Results:** The median follow-up was 44.9 months. Each one-point increase in the baseline PNI was independently associated with lower hazards of progression (HR 0.952, 95% CI 0.923–0.983) and death (HR 0.919, 95% CI 0.884–0.956), corresponding to HR 0.782 (95% CI 0.670–0.918) and HR 0.656 (95% CI 0.540–0.799) per five-point increase. For overall survival, in formal comparisons on identical patient sets, neither the NLR nor the SIRI added prognostic information to a model containing the PNI (likelihood ratio *p* = 0.383 and *p* = 0.335), whereas the PNI added information to models containing either ratio (*p* < 0.001 and *p* = 0.004). In exploratory landmark analyses, change in the PNI added little to the baseline value; apparent associations between increases in the NLR or SIRI by cycle 3 and overall survival did not persist after winsorisation of extreme change scores. **Conclusions:** Baseline PNI, analysed as a continuous variable and without a data-derived threshold, was independently associated with progression-free and overall survival in this cohort. For overall survival, neither baseline inflammatory ratio added detectable prognostic information beyond the index, and repeated measurement during treatment added little to the baseline value, although the confidence intervals do not exclude modest effects. Because all patients received a CDK4/6 inhibitor and no comparator arm was available, these findings support a prognostic rather than a predictive interpretation, and external validation is required before the index can inform clinical decisions.

## 1. Introduction

Cyclin-dependent kinase 4/6 (CDK4/6) inhibitors combined with endocrine therapy are the standard of care for hormone receptor–positive, human epidermal growth factor receptor 2 (HER2)-negative metastatic breast cancer [1,2,3]. Outcomes within this population vary widely: some patients progress within a few months, whereas others remain on treatment for several years. Molecular predictors of this variation have not entered routine practice, and the assays proposed require tumour material and infrastructure that are frequently unavailable when treatment decisions are made [4]. Markers derived from routine pretreatment laboratory tests would therefore be clinically valuable.

Host nutritional and inflammatory status is one candidate, with a rationale specific to this treatment context. CDK4/6 inhibition enhances tumour antigen presentation, suppresses regulatory T-cell proliferation and augments cytotoxic T-cell activation [5,6]; if the activity of these agents depends in part on host immune competence, pretreatment host status may carry prognostic information relevant to this therapy in particular. The prognostic nutritional index (PNI) combines serum albumin and the absolute lymphocyte count [7] and therefore reflects nutritional reserve and adaptive immune capacity simultaneously, whereas the neutrophil-to-lymphocyte ratio (NLR) and the systemic inflammation response index (SIRI) capture the inflammatory dimension alone. Because all three share the lymphocyte count as a component, it remains unclear whether they convey distinct prognostic information.

Evidence for the PNI in patients receiving CDK4/6 inhibitors remains limited; the two largest reports are retrospective cohorts, both reporting longer survival in patients with a higher index: one of 431 patients, in whom a low PNI was associated with shorter progression-free and overall survival [8], and one of 106 patients, in whom overall survival was longer in the high-PNI group, although response rates did not differ significantly [9]. In both cohorts the index was measured once before the first dose. The findings for the purely inflammatory ratios are less consistent. A high baseline NLR was associated with shorter progression-free and overall survival in a real-world cohort of 2218 patients [10], and with shorter progression-free survival in the 81 patients treated with a CDK4/6 inhibitor within a cohort of 97, in whom the association with overall survival was confined to the 48 patients treated in the first line [11]; the NLR was not associated with survival in a cohort of 38, in whom the C-reactive protein-to-albumin ratio was the only index independently associated with overall survival [12].

Several methodological features constrain the interpretation of these findings. Cohorts have been heterogeneous with respect to treatment line and endocrine partner. Thresholds were derived from the cohorts themselves by median split or receiver operating characteristic analysis; reported cut-offs for the PNI in breast cancer vary appreciably between studies [9,13,14] by a margin difficult to attribute to biology, and data-derived dichotomisation tends to inflate effect estimates and limits transferability between populations. Multivariable adjustment has been inconsistent, with covariates often selected based on univariable *p* values rather than clinical reasoning. To our knowledge, the PNI has not been compared directly with inflammatory ratios within a single CDK4/6 inhibitor-treated cohort, so it is not known whether they identify the same patients. It therefore remains unclear whether the reported association between a low PNI and poor outcome reflects a robust prognostic signal or the analytical choices used to demonstrate it.

A further consideration concerns timing. Nutritional and immune status are not fixed attributes: albumin and the lymphocyte count both alter during treatment, partly through the myelosuppressive effect of CDK4/6 inhibition and partly through the evolving relationship between host and tumour. In the neoadjuvant setting a fall in the PNI during chemotherapy has been reported to predict worse outcome [15], raising the possibility that a measurement repeated early during treatment, or its change from the pretreatment value, carries information that a single determination does not. Whether this holds during CDK4/6 inhibitor therapy is unknown, and the authors of the largest series identified dynamic monitoring as a question for future work [8].

We therefore examined the baseline PNI in a consecutive single-centre cohort of patients treated with a CDK4/6 inhibitor and endocrine therapy. The primary objective was to determine whether the baseline PNI, modelled as a continuous variable and without any threshold derived from the present data, is independently associated with progression-free and overall survival after adjustment for a clinically selected covariate set. Two secondary objectives followed. The first was to establish whether the NLR and the SIRI added prognostic information beyond the PNI when examined in the same patients. The second was to determine whether the index measured before the second and third cycles, or its change from the pretreatment value, was associated with outcome once the baseline measurement had been accounted for. Rather than providing further evidence that the PNI is prognostic, the study tested whether that association persists under a more conservative analytical approach.

## 2. Methods

### 2.1. Study Design

This was a single-centre, retrospective cohort study conducted in a real-world setting. We reviewed consecutive patients with hormone receptor–positive, HER2-negative metastatic breast cancer who received a CDK4/6 inhibitor in combination with endocrine therapy at the Marmara University School of Medicine, Istanbul, Türkiye, between June 2020 and May 2025. The data cut-off for the present analysis was 31 July 2026.

The institutional ethics committee (Marmara University School of Medicine, Ethics Committee for Non-Drug and Non-Medical Device Research; protocol code 09.2025.25-1093; approved 19 December 2025) approved the study, and written informed consent was waived in accordance with national legislation and institutional standards. The study was conducted in accordance with the Declaration of Helsinki and is reported in line with the Strengthening the Reporting of Observational Studies in Epidemiology (STROBE) statement [16] and the Reporting Recommendations for Tumour Marker Prognostic Studies (REMARK) [17].

### 2.2. Patient Selection

All consecutive patients meeting the eligibility criteria during the study period were included to minimise selection bias. Eligible patients were women aged 18 years or older with histologically confirmed hormone receptor–positive, HER2-negative metastatic breast cancer who received ribociclib or palbociclib together with an aromatase inhibitor or fulvestrant at any line of therapy for metastatic disease. Documented follow-up allowing assessment of disease status was required. Pretreatment complete blood count and serum albumin values obtained before the first dose of CDK4/6 inhibitor were abstracted where available; patients in whom a value needed to calculate the PNI was missing were retained in the descriptive analyses and excluded from analyses involving the index.

One patient had inoperable locoregional recurrence without documented distant metastasis and was treated within the same pathway; this patient was retained in the cohort. Because this single case does not fully conform to the definition of metastatic disease, both primary multivariable models were repeated with this patient excluded as a sensitivity analysis (Supplementary Table S10). Patients with a concurrent or previous second primary malignancy and those in whom outcome status could not be ascertained were excluded. No further exclusion criteria were applied; in particular, no minimum follow-up or treatment exposure duration was required.

Of 193 patients identified, one was excluded because disease status could not be determined, leaving 192 patients in the analysis cohort. The baseline PNI could not be calculated in two patients because albumin or lymphocyte values were missing; these patients were included in descriptive analyses and excluded from analyses involving the PNI.

### 2.3. Data Collection

Clinical, pathological, laboratory and treatment data were extracted from the institutional electronic medical records. Clinical variables comprised age at initiation of CDK4/6 inhibitor therapy, menopausal status, height and weight (from which body mass index was derived), comorbidities and concomitant medications. Comorbidities were coded individually and summarised as a count; polypharmacy was defined as the concurrent use of five or more medications.

Pathological variables included histological subtype, hormone receptor and HER2 status, Ki-67 labelling index and histological grade. HER2-negative status was defined according to the American Society of Clinical Oncology/College of American Pathologists (ASCO/CAP) criteria in force at the time of diagnosis [18]. Oestrogen and progesterone receptor positivity were defined as nuclear staining in at least 1% of invasive tumour cells by immunohistochemistry, in accordance with the ASCO/CAP criteria in force at the time of diagnosis. Cases scoring 2+ by immunohistochemistry were classified as HER2-negative on the basis of in situ hybridisation performed as part of routine diagnostic practice. Immunohistochemical HER2 scores were recorded, allowing the proportion of tumours meeting the current definition of HER2-low expression to be reported. Histological grade was available in 96 patients (50.0%); its distribution among evaluable cases is reported in Supplementary Table S12, and grade was not entered into any regression model. Histological subtype was classified according to current World Health Organization terminology, and tumours previously designated invasive ductal carcinoma are reported here as invasive breast carcinoma of no special type (NST). The heterogeneous “other” category comprised mucinous carcinoma (*n* = 7), mixed ductal and mucinous carcinoma (*n* = 3), mixed ductal and lobular carcinoma (*n* = 8) and invasive micropapillary carcinoma (*n* = 2); in three cases the available pathology records did not permit more specific histological subclassification. Pathological variables were abstracted from routine institutional diagnostic reports; the Ki-67 labelling index was taken directly from those reports and was not re-scored for the present study, and no central pathological review was undertaken. Institutional practice is to obtain a biopsy of a metastatic lesion at recurrence when visceral or nodal disease is accessible and to reassess receptor status on that specimen. In patients with bone-only disease, biopsy of a skeletal lesion is not undertaken routinely, in part because decalcification degrades immunohistochemical assessment; the values recorded for these patients therefore derive from the primary tumour. Because a substantial proportion of the cohort had bone-only disease with or without nodal involvement, the source of the pathological data is heterogeneous in this respect. The proportion of patients in whom receptor status was formally reassessed on a metastatic specimen was not captured in the study database and could not be reconstructed reliably.

Treatment variables comprised the CDK4/6 inhibitor administered, the endocrine partner, the line of therapy in the metastatic setting, prior adjuvant endocrine therapy, and dose reductions with their indications. Metastatic involvement was recorded separately for each organ site (bone, lymph node, lung, liver, brain, peritoneum, adrenal gland), allowing liver involvement to be examined independently of other visceral sites. Visceral disease was defined as involvement of the liver, lung, peritoneum, adrenal gland or brain; bone and lymph node involvement were not counted as visceral. Bone-only disease was defined as skeletal involvement, with or without lymph node metastasis, in the absence of any visceral involvement. The two categories are mutually exclusive but do not account for the whole cohort, since eight patients had disease at sites not covered by either category: lymph node involvement in six, of whom two had an additional deposit at a site outside the visceral definition used here; skin in one; and inoperable locoregional recurrence in one.

Laboratory variables were obtained from the complete blood count and biochemistry panel performed immediately before the first dose of CDK4/6 inhibitor and comprised haemoglobin, absolute neutrophil, lymphocyte and monocyte counts, platelet count and serum albumin. Serum albumin was reported in g/L and leucocyte subpopulations in cells/µL.

Best radiological response was categorised as complete response, partial response, stable disease or progressive disease. Response in patients with measurable soft-tissue disease was assessed according to the Response Evaluation Criteria in Solid Tumours (RECIST) version 1.1 [19]. In patients whose disease was confined to bone, response was assessed on ^18^F-fluorodeoxyglucose positron emission tomography/computed tomography (-FDG PET/CT) in accordance with routine institutional practice: resolution of FDG uptake in lesions that were avid at baseline was recorded as complete response, and a reduction in FDG uptake or sclerotic transformation of previously lytic lesions was recorded as partial response. Because bone lesions are not considered measurable under RECIST version 1.1, response rates in this cohort are not directly comparable with those of trials restricted to patients with measurable disease. The date of initiation of CDK4/6 inhibitor therapy, the date of documented disease progression, the date of last contact and the date of death were recorded for every patient.

### 2.4. Biomarker Calculation

All indices were derived from the pretreatment laboratory values defined above. The PNI was calculated according to the formula of Onodera et al. [7] as 10 × serum albumin (g/dL, converted from the reported g/L) + 0.005 × total lymphocyte count (/µL). The NLR was calculated as the absolute neutrophil count divided by the absolute lymphocyte count, and the SIRI was calculated as (absolute neutrophil count × absolute monocyte count)/absolute lymphocyte count, with all counts expressed in ×10^9^/L. The PNI was the primary biomarker of interest; the NLR and SIRI were examined as comparators.

### 2.5. Outcomes

The primary endpoint was progression-free survival, defined as the interval from the first dose of CDK4/6 inhibitor to radiologically documented disease progression or death from any cause, whichever occurred first. Overall survival, defined as the interval from the first dose of CDK4/6 inhibitor to death from any cause, was the secondary endpoint. We also assessed the institutionally assessed best response rate and the disease control rate. Because response in patients with bone-only disease was determined on ^18^F-FDG PET/CT rather than by anatomical measurement, this endpoint is not equivalent to a conventional RECIST-defined objective response rate and is designated accordingly throughout. Best response is reported separately for patients with measurable soft-tissue disease, for those with bone-only disease, and for those with other non-measurable disease (Table 1).

Disease assessments were performed as part of routine institutional clinical practice, at intervals determined by the treating physician, and were not subject to central radiological review. The date of progression was taken as the date of the imaging study on which progression was first demonstrated. Patients without progression were censored at the date of the last assessment documenting absence of progression. For overall survival, we verified vital status for every patient through institutional records and the national death registration system; patients confirmed alive were censored at the common data cut-off of 31 July 2026.

## 3. Statistical Analysis

### 3.1. Descriptive Statistics

We examined the distribution of continuous variables using the Shapiro–Wilk test. Except for age, all continuous variables deviated significantly from normality; for consistency, we present continuous variables as median with interquartile range (IQR) throughout and categorical variables as counts and percentages. We quantified associations between the three indices and between each index and its constituent laboratory values using the Spearman rank correlation coefficients.

### 3.2. Survival Analysis

We estimated survival distributions using the Kaplan–Meier method and compared them with the log-rank test. The median follow-up was estimated by the reverse Kaplan–Meier method, in which patients who died were censored and those alive at last contact were treated as events. Hazard ratios (HRs) with 95% confidence intervals (CIs) were derived from the Cox proportional hazards regression models. All models were fitted using the enter method; stepwise selection was not used. All tests were two-sided, and a *p* value below 0.05 was considered statistically significant. Analyses were performed with IBM SPSS Statistics, version 27.0 (IBM Corp., Armonk, NY, USA), except for the restricted cubic spline models, and the Schoenfeld residual tests, which were performed in R version 4.6.1 (R Foundation for Statistical Computing, Vienna, Austria) using the survival package. For consistency between the two packages, the Breslow approximation for tied event times was used throughout. A generative artificial intelligence tool (Claude, Anthropic, Opus 4.5) was used during manuscript preparation for language editing, clarity and phrasing, and during peer-review revision to assist in drafting and checking the R code for the restricted cubic spline and Schoenfeld residual analyses. The code was executed by the authors, and all AI-assisted text and statistical outputs were independently reviewed, checked and interpreted by the authors, who take full responsibility for the reported findings. The R code is available from the corresponding author on request. All other analyses were performed by the authors in IBM SPSS Statistics.

### 3.3. Modelling of the PNI

The PNI was analysed as a continuous variable in all regression models, and hazard ratios are reported per one-point increase and, to aid clinical interpretation, additionally per five-point increase. Reported thresholds for the index vary considerably across breast cancer series, and deriving a cut-off from the present cohort would risk overstating the effect size and yielding a threshold that does not transfer to other populations. For graphical presentation only, patients were dichotomised at a value of 45, a threshold previously reported in the breast cancer literature [14]; this dichotomisation was not derived from these data, was not used in any regression model, and is presented solely to visualise the survival distributions. The analysis of the PNI as a continuous variable constitutes the principal evidence of this study.

### 3.4. Variable Selection for Multivariable Models

Baseline PNI was the variable of primary interest. We constructed the primary multivariable models for progression-free survival and overall survival with the same covariate set, comprising PNI together with age, liver metastasis, and line of therapy. We selected these three covariates on clinical grounds rather than based on univariable *p* values: line of therapy and hepatic involvement are established determinants of outcome in metastatic breast cancer, and age was included as a standard demographic adjustment. Age was retained despite showing no univariable association with either endpoint, and several variables that did reach univariable significance were not entered, for the reasons set out below. Using an identical covariate set for both endpoints allows direct comparison of the two models.

Visceral metastasis and bone-only disease were not entered alongside liver metastasis because these categories describe overlapping patterns of metastatic distribution. We excluded the number of involved metastatic organs from the primary models for the same reason, since hepatic involvement contributes to that count; we instead examined it in a separate sensitivity analysis, in which the estimate for liver metastasis was attenuated, consistent with the two variables carrying partly the same information. The Ki-67 index was not included in the primary models because it was missing in 19 patients, and histological grade was not modelled because it was available in only half of the cohort.

### 3.5. Supplementary Models

Seven additional models were fitted. The first repeated the primary progression-free survival model, adding the Ki-67 index in the subgroup for whom this variable was available. The second and third added the SIRI and the NLR, respectively, to the primary overall survival model. We examined the two inflammatory indices in separate models rather than together, since both are constructed from neutrophil and lymphocyte counts and therefore convey overlapping information. The fourth and fifth added the number of involved metastatic organs to each primary model to examine whether the association with the PNI could be accounted for by tumour burden. The sixth and seventh models added the CDK4/6 inhibitor administered and the endocrine partner to each primary model to examine whether treatment selection could explain the association. These analyses are presented in the Supplementary Materials. Because the loss of statistical significance of one variable in the presence of another does not by itself establish that the two differ in prognostic content, the models containing the PNI and those containing an inflammatory index were also compared formally. For each inflammatory index we fitted three models on an identical patient set—the covariate set with the PNI alone, with the inflammatory index alone, and with both—and compared them by likelihood ratio tests in both directions, each with one degree of freedom, reporting the Akaike information criterion (AIC) alongside. Comparisons involving the NLR were restricted to the 190 patients with an evaluable baseline PNI and those involving the SIRI to the 187 patients with an evaluable monocyte count, so that all models within a comparison were fitted on the same observations. Because the PNI is a fixed linear combination of its two constituents, we also examined whether the composite index conveyed information beyond either component alone. Expressed in the units recorded here, the index equals serum albumin in g/L plus 0.005 times the lymphocyte count per µL, so that one index point corresponds to 1 g/L of albumin or to 200 lymphocytes/µL. We therefore fitted the primary covariate set with the PNI, with albumin alone, with the lymphocyte count alone expressed per 200 cells/µL, and with both components entered with freely estimated coefficients. The model containing the PNI is nested within the model containing both components. A likelihood ratio test with one degree of freedom therefore examines whether the fixed weighting of the index is consistent with these data, and two further tests examine whether each component adds information beyond the other.

### 3.6. Serial Measurements of the PNI

The PNI, the NLR and the SIRI were recalculated from the blood counts obtained before cycles 2 and 3, and change was expressed as the difference from the baseline value. The serial analyses of the NLR and the SIRI were exploratory and are reported for completeness, since the same measurements were available for all three indices. These analyses were restricted to the 183 patients with complete blood count and serum albumin available at all three time points. Because a patient must remain on treatment to contribute a cycle 3 measurement, survival time in these analyses was measured from a landmark set at two months, which corresponds to the point at which the cycle 3 blood count is taken under a 28-day cycle; patients with an event before the landmark were excluded, and the remaining patients were followed from that point. We applied the same two-month landmark to the change to cycle 2 so that both change measures were evaluated within the same landmark cohort and could be compared directly; survival follow-up for both analyses began at the landmark. Change scores are negatively correlated with the baseline value by construction, so every model containing a change term also contained the corresponding baseline value, and the hazard ratio reported for change is therefore the association remaining after accounting for the baseline value. We fitted these models in two specifications: first with the baseline value as the only other term and then with the covariate set used in the primary models added. These analyses were secondary and were not used to modify the primary models. Because the change scores for the NLR and the SIRI had wide and asymmetrical distributions, the corresponding cycle 3 models were repeated after winsorising each change score at its 5th and 95th percentiles (Supplementary Table S11).

### 3.7. Assumptions and Missing Data

We assessed the proportional hazards assumption graphically by inspecting log-minus-log survival plots for the categorical covariates (Supplementary Figure S1). The assumption was also assessed formally using scaled Schoenfeld residuals and global Schoenfeld tests. We also examined the assumption of a linear relationship between the continuous PNI and the log hazard using restricted cubic splines. Three knots were placed at the 10th, 50th and 90th percentiles of the observed distribution. Three knots were chosen to limit model flexibility given the number of events, particularly for overall survival. With three knots the spline adds a single non-linear term, so departure from linearity was tested by a likelihood ratio test with one degree of freedom comparing the spline model with the corresponding linear model.

Missing data were not imputed, and all analyses were performed on complete cases. Missing values were infrequent for the variables in the primary models: the PNI could not be calculated in two patients, and age, liver metastasis and line of therapy were complete for the entire cohort. We therefore fitted the primary multivariable models in 190 patients. The number of patients and events contributing to each analysis is reported in the corresponding table.

### 3.8. Exploratory Analyses

The indices examined in this study were the PNI, the NLR and the SIRI. No other index derived from routine blood counts was calculated or tested, and the results for all three are reported here. The PNI was designated the index of primary interest because it incorporates a nutritional component that is absent from the purely cell-based ratios and because it has an established literature in solid tumours. This designation was made after the data had been assembled rather than in a prespecified statistical analysis plan, and the findings should therefore be regarded as hypothesis-generating.

## 4. Results

### 4.1. Patient Characteristics

A total of 192 patients with hormone receptor–positive, HER2-negative metastatic breast cancer who received CDK4/6 inhibitor therapy were analysed. Table 1 summarises the baseline characteristics. The median age was 58.1 years (IQR 48.7–68.5), and 139 patients (72.4%) were postmenopausal. Ribociclib was used in 143 patients (74.5%) and palbociclib in 49 (25.5%); therapy was given in the first-line setting in 129 patients (67.2%). Bone was the most common metastatic site (85.4%), and liver metastases were present in 48 patients (25.0%). At least one comorbidity was recorded in 132 patients (68.8%). The median baseline PNI was 51.0 (IQR 48.0–54.1). An institutionally assessed best response was achieved in 146 patients (76.0%) and disease control in 168 (87.5%). Best response was recorded in 72 of 98 patients with measurable soft-tissue disease assessed by RECIST 1.1 (73.5%), in 69 of 85 patients with bone-only disease assessed by ^18^F-FDG PET/CT (81.2%) and in five of nine patients with other non-measurable disease (55.6%); the distribution of best response did not differ significantly between these categories (χ^2^ = 9.81, df = 6, *p* = 0.133). The corresponding disease control rates were 82.7%, 92.9% and 88.9%.

### 4.2. Survival Outcomes

At a data cut-off of 31 July 2026, disease progression had occurred in 124 patients (64.6%), and 83 patients (43.2%) had died. The median follow-up, estimated by the reverse Kaplan–Meier method, was 44.9 months (95% CI 41.2–48.7). The median progression-free survival was 24.7 months (95% CI 18.1–31.3), and the median overall survival was 49.1 months (95% CI 37.5–60.6).

**Figure 1:**
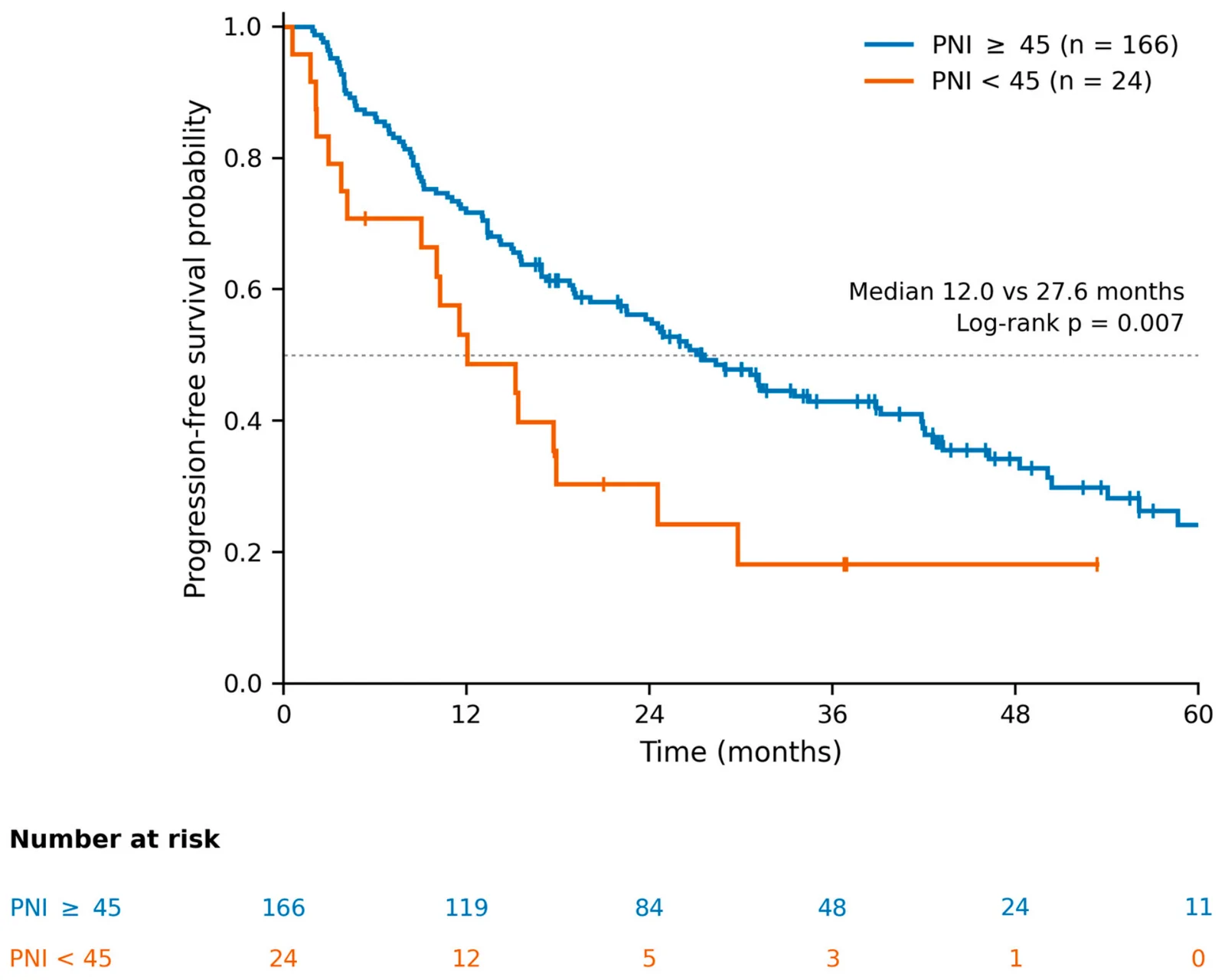
Kaplan–Meier estimates of progression-free survival according to baseline prognostic nutritional index (PNI). Patients were dichotomised at a PNI of 45 (PNI < 45, *n* = 24; PNI ≥ 45, *n* = 166). The vertical ticks denote censored observations, and the dashed horizontal line marks 50% survival. The numbers of patients at risk are shown below the plot. Baseline PNI could be calculated in 190 of the 192 patients because albumin and lymphocyte counts were missing in 2.

**Figure 2:**
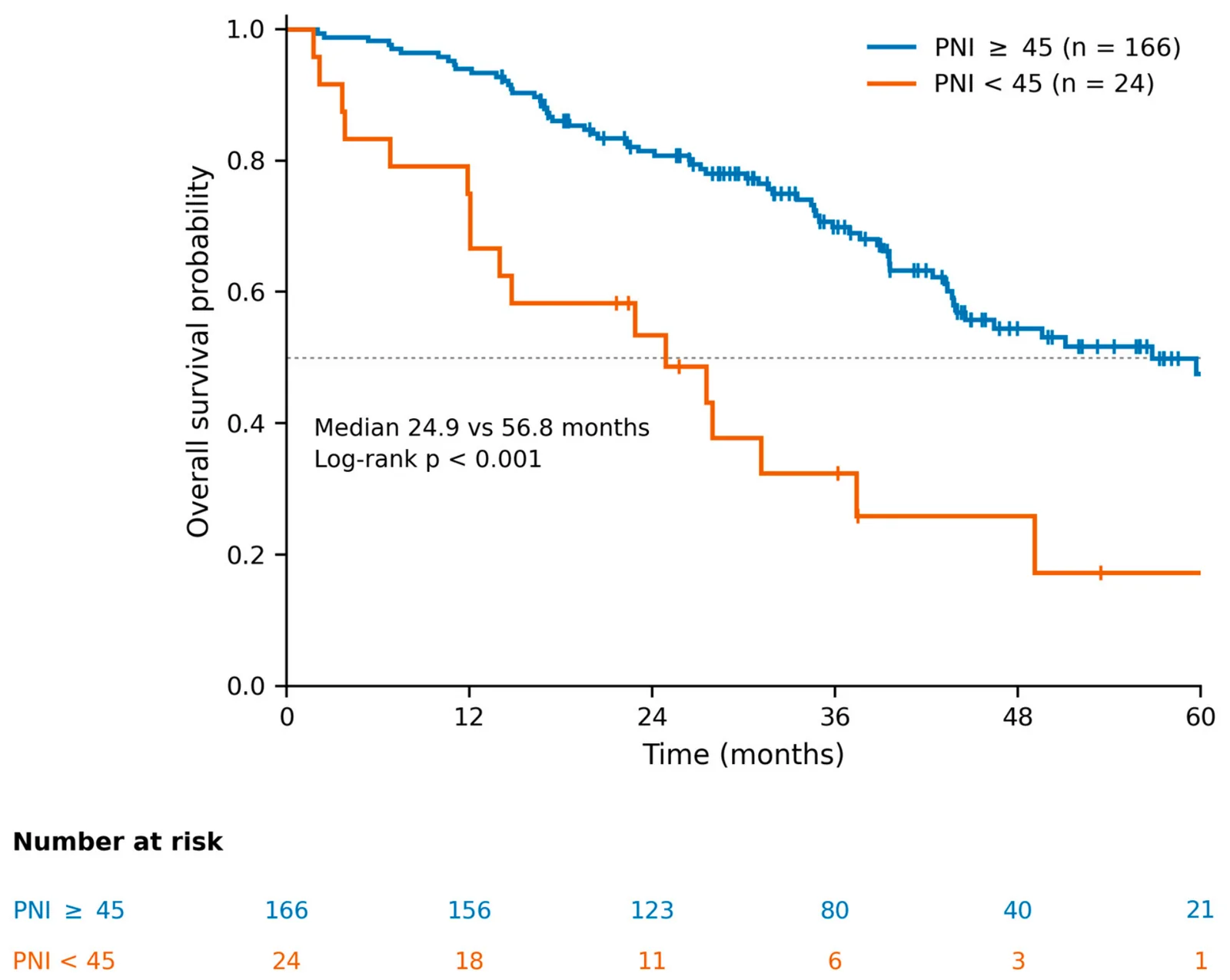
Kaplan–Meier estimates of overall survival according to baseline prognostic nutritional index (PNI). Patients were dichotomised at a PNI of 45 (PNI < 45, *n* = 24; PNI ≥ 45, *n* = 166). The vertical ticks denote censored observations, and the dashed horizontal line marks 50% survival. Neither bound of the confidence interval for the median could be estimated in the PNI ≥ 45 group, as the survival curve reached 50% only toward the end of follow-up, when few patients remained at risk. The numbers of patients at risk are shown below the plot.

For graphical presentation, patients were dichotomised at a PNI of 45, a threshold previously reported in the literature. The baseline PNI could be calculated for 190 of 192 patients; of these, 24 (12.6%) had a PNI below 45, and 166 (87.4%) had a PNI of 45 or higher. The median progression-free survival was 12.0 months (95% CI 4.4–19.6) in patients with a PNI below 45 and 27.6 months (95% CI 22.1–33.1) in those with a PNI of 45 or higher (log-rank *p* = 0.007; Figure 1). The median overall survival was 24.9 months (95% CI 7.5–42.3) and 56.8 months (95% CI not estimable), respectively (log-rank *p* < 0.001; Figure 2).

### 4.3. Correlation Between the Indices

The three indices were only moderately related. The PNI correlated inversely with the NLR (Spearman rho −0.49) and weakly with the SIRI (−0.26), whereas the NLR and the SIRI were closely correlated with each other (0.80). The pattern of correlation with the constituent laboratory values differed accordingly: the PNI tracked albumin (0.74) and the lymphocyte count (0.68) but was unrelated to the neutrophil count (−0.04, *p* = 0.578), whereas the NLR and the SIRI tracked the neutrophil count (0.72 and 0.79) and, for the SIRI, the monocyte count (0.72); the correlation between the SIRI and the lymphocyte count was not significant (−0.10, *p* = 0.157). The full correlation matrix is given in Supplementary Table S1.

### 4.4. Progression-Free Survival

Univariable Cox regression analyses are shown in Table 2. A higher baseline PNI was associated with a lower hazard of progression (HR 0.946, 95% CI 0.917–0.977, *p* < 0.001). Liver metastasis (HR 2.414, 95% CI 1.661–3.509, *p* < 0.001) and each successive line of therapy (HR 1.239, 95% CI 1.105–1.389, *p* < 0.001) were associated with a higher hazard of progression, as were visceral metastasis (HR 1.684, 95% CI 1.177–2.408, *p* = 0.004), each additional metastatic organ (HR 1.632, 95% CI 1.318–2.021, *p* < 0.001) and the Ki-67 index (HR 1.014, 95% CI 1.002–1.026, *p* = 0.023), whereas bone-only disease was associated with a lower hazard (HR 0.650, 95% CI 0.453–0.934, *p* = 0.020). Fulvestrant as the endocrine partner was also associated with a higher hazard (HR 1.561, 95% CI 1.089–2.237, *p* = 0.015), although this is likely to reflect its preferential use in later lines. Lung metastasis, bone metastasis, the CDK4/6 inhibitor administered, NLR and SIRI were not associated with progression-free survival.

In the multivariable model, each 1-point increase in the baseline PNI was associated with a ~5% lower hazard of progression (HR 0.952, 95% CI 0.923–0.983, *p* = 0.003); expressed per five-point increase, this corresponds to a hazard ratio of 0.782 (95% CI 0.670–0.918). Liver metastasis (HR 2.114, 95% CI 1.421–3.146, *p* < 0.001) and line of therapy (HR 1.251, 95% CI 1.106–1.416, *p* < 0.001) remained independently associated with progression-free survival; age was not (HR 0.999, 95% CI 0.984–1.014, *p* = 0.917). Excluding the single patient with inoperable locoregional recurrence did not materially alter these estimates (*n* = 189; HR 0.953, 95% CI 0.923–0.983, *p* = 0.003; Supplementary Table S10).

Because Ki-67 data were unavailable for 19 patients, we excluded Ki-67 from the primary model. We fitted a supplementary model adjusted for Ki-67, presented in Supplementary Table S2. In that model (*n* = 171; 112 events), the baseline PNI remained independently associated with progression-free survival (HR 0.954, 95% CI 0.923–0.986, *p* = 0.005), whereas Ki-67 was not (HR 1.009, 95% CI 0.997–1.022, *p* = 0.135).

### 4.5. Overall Survival

Univariable analyses of overall survival are shown in Table 3. A higher baseline PNI was associated with a lower hazard of death (HR 0.911, 95% CI 0.878–0.946, *p* < 0.001). Liver metastasis (HR 2.436, 95% CI 1.572–3.776, *p* < 0.001), visceral metastasis (HR 1.811, 95% CI 1.157–2.835, *p* = 0.009), line of therapy (HR 1.186, 95% CI 1.019–1.381, *p* = 0.028), the number of metastatic organs (HR 1.412, 95% CI 1.104–1.806, *p* = 0.006), SIRI (HR 1.194, 95% CI 1.072–1.330, *p* = 0.001), and NLR (HR 1.034, 95% CI 1.006–1.062, *p* = 0.015) were also associated with overall survival, whereas bone-only disease was associated with a lower hazard of death (HR 0.598, 95% CI 0.379–0.943, *p* = 0.027). Ki-67 was not associated with overall survival (HR 1.000, 95% CI 0.985–1.015, *p* = 0.973).

In the primary multivariable model, each 1-point increase in baseline PNI was associated with an approximately 8% lower hazard of death (HR 0.919, 95% CI 0.884–0.956, *p* < 0.001); expressed per five-point increase, this corresponds to a hazard ratio of 0.656 (95% CI 0.540–0.799). Liver metastasis also remained independently associated with outcome (HR 2.060, 95% CI 1.289–3.293, *p* = 0.003), whereas line of therapy (HR 1.145, 95% CI 0.972–1.350, *p* = 0.105) and age (HR 1.007, 95% CI 0.988–1.026, *p* = 0.481) did not. Excluding the single patient with inoperable locoregional recurrence did not materially alter these estimates (*n* = 189; HR 0.919, 95% CI 0.884–0.956, *p* < 0.001; Supplementary Table S10).

In two further models, SIRI and NLR were each added separately to this covariate set; the two indices were not entered together because both are derived from the neutrophil and lymphocyte counts and therefore convey overlapping information. After adjustment, neither SIRI (HR 1.076, 95% CI 0.931–1.243, *p* = 0.322) nor NLR (HR 1.019, 95% CI 0.981–1.058, *p* = 0.331) remained associated with outcome, while the estimate for the baseline PNI was little changed in both models (HR 0.933, 95% CI 0.890–0.979, *p* = 0.005 and HR 0.925, 95% CI 0.887–0.965, *p* < 0.001, respectively; Supplementary Tables S3 and S4). In formal comparisons on identical patient sets, adding the NLR to a model already containing the PNI did not improve model fit (likelihood ratio χ^2^ = 0.761, df = 1, *p* = 0.383), nor did adding the SIRI (χ^2^ = 0.929, df = 1, *p* = 0.335). The reverse comparisons were significant: adding the PNI to a model containing the NLR improved fit (χ^2^ = 12.279, df = 1, *p* < 0.001), as did adding it to a model containing the SIRI (χ^2^ = 8.164, df = 1, *p* = 0.004). The Akaike information criterion was lowest for the models containing the PNI alone (741.1 versus 752.7 for the NLR and 722.7 versus 729.9 for the SIRI; Supplementary Table S8). Analyses of the components of the index gave different results for the two endpoints (Supplementary Table S9). The fixed Onodera weighting was consistent with the data for both progression-free survival (likelihood ratio χ^2^ = 1.245, df = 1, *p* = 0.265) and overall survival (χ^2^ = 0.014, df = 1, *p* = 0.906). For overall survival, both constituents contributed independently (albumin HR 0.921, 95% CI 0.877–0.966, *p* < 0.001; lymphocyte count per 200 cells/µL HR 0.916, 95% CI 0.854–0.982, *p* = 0.014). Each added information beyond the other: χ^2^ = 6.455, *p* = 0.011 for the lymphocyte count added to albumin and χ^2^ = 9.505, *p* = 0.002 for albumin added to the lymphocyte count. The model containing the composite index gave the lowest Akaike information criterion of the four specifications (741.1 versus 747.6 for albumin alone and 750.6 for the lymphocyte count alone). For progression-free survival the pattern differed: albumin alone accounted for the association (HR 0.936, 95% CI 0.900–0.974, *p* = 0.001), the lymphocyte count added no further information (χ^2^ = 0.995, df = 1, *p* = 0.319), and the model containing albumin alone fitted marginally better than the composite index (AIC 1108.6 versus 1108.8).

Because tumour burden might account for the association, a further sensitivity analysis added the number of involved organs to each primary model. The estimate for the baseline PNI was essentially unchanged for progression-free survival (HR 0.956, 95% CI 0.926–0.987, *p* = 0.006) and for overall survival (HR 0.920, 95% CI 0.885–0.956, *p* < 0.001); the number of involved organs remained associated with progression-free survival (HR 1.293, 95% CI 1.008–1.657, *p* = 0.043) but not with overall survival (HR 1.065, 95% CI 0.792–1.433, *p* = 0.676) (Supplementary Table S5).

A further sensitivity analysis added the CDK4/6 inhibitor administered and the endocrine partner to each primary model. The estimate for the baseline PNI was unchanged, as were the estimates for progression-free survival (HR 0.953, 95% CI 0.922–0.985, *p* = 0.005) and overall survival (HR 0.916, 95% CI 0.880–0.955, *p* < 0.001). Neither the agent administered nor the endocrine partner was independently associated with either endpoint in these models (Supplementary Table S6).

### 4.6. Model Assumptions

Tests based on scaled Schoenfeld residuals provided no statistically significant evidence of non-proportionality for the baseline PNI or for any other covariate in either primary model; the global tests were also non-significant (progression-free survival, *p* = 0.657; overall survival, *p* = 0.167; Supplementary Table S13). Diagnostic plots for the baseline PNI showed no systematic time-dependent pattern (Supplementary Figure S2). Restricted cubic splines with knots at 43.0, 51.0 and 57.6 likewise provided no evidence of departure from linearity in the relationship between the index and the log hazard, either for progression-free survival (likelihood ratio χ^2^ = 0.355, df = 1, *p* = 0.551) or for overall survival (χ^2^ = 0.867, df = 1, *p* = 0.352). These results do not establish that the assumptions hold, and the hazard ratios are accordingly interpreted as average effects over the follow-up period.

### 4.7. Serial PNI Measurements

Serial measurements were available in 183 patients. The median PNI was 51.0 (IQR 48.0–54.0) at baseline, 49.5 (IQR 46.0–53.5) before cycle 2 and 49.5 (IQR 46.5–53.0) before cycle 3. The median change from baseline was −1.5 by cycle 2 (IQR −4.5 to 1.5; range −13.5 to 12.5) and −1.5 by cycle 3 (IQR −4.0 to 1.5; range −19.5 to 10.0). In landmark analyses beginning at two months, the PNI was associated with both endpoints at each of the three time points, and the association was strongest for the baseline value. For progression-free survival, the hazard ratio per one-point increase was 0.954 (95% CI 0.923–0.986, *p* = 0.005) at baseline, 0.963 (95% CI 0.935–0.993, *p* = 0.015) before cycle 2 and 0.961 (95% CI 0.931–0.991, *p* = 0.011) before cycle 3. For overall survival, the corresponding estimates were 0.917 (95% CI 0.882–0.954), 0.935 (95% CI 0.903–0.968) and 0.938 (95% CI 0.903–0.974), all *p* < 0.001. When change from baseline was entered together with the baseline value, no change term was associated with either endpoint. For progression-free survival, the hazard ratio for the change to cycle 2 was 0.986 (95% CI 0.946–1.029, *p* = 0.527), and for the change to cycle 3 it was 0.982 (95% CI 0.940–1.025, *p* = 0.399). For overall survival, the corresponding estimates were 0.973 (95% CI 0.926–1.023, *p* = 0.284) and 0.984 (95% CI 0.934–1.037, *p* = 0.558). When the covariate set used in the primary models was added, the change to cycle 3 reached nominal significance for progression-free survival (HR 0.950, 95% CI 0.907–0.994, *p* = 0.028), whereas the change to cycle 2 did not (HR 0.977, 95% CI 0.935–1.022, *p* = 0.311), and neither change term was associated with overall survival (HR 0.961, 95% CI 0.913–1.013, *p* = 0.138 and HR 0.969, 95% CI 0.918–1.022, *p* = 0.250) (Supplementary Table S7). We repeated the same analyses for the NLR and the SIRI, for which serial measurements were also available. In models adjusted for the baseline value and the primary covariate set, an increase by cycle 3 was associated with overall survival for both the NLR (HR 1.134, 95% CI 1.038–1.239, *p* = 0.005) and the SIRI (HR 1.443, 95% CI 1.086–1.919, *p* = 0.012), whereas neither change to cycle 2 was associated with either endpoint, and neither change term was associated with progression-free survival (Supplementary Table S7). The distributions of these change scores were wide and asymmetrical (range −14.2 to +20.9 for the NLR and −6.34 to +3.95 for the SIRI), and the estimates were highly sensitive to a small number of extreme increases. After winsorisation at the 5th and 95th percentiles, neither association persisted: the hazard ratio for the change in the NLR to cycle 3 fell to 0.989 (95% CI 0.839–1.166, *p* = 0.897) and that for the SIRI to 1.111 (95% CI 0.853–1.446, *p* = 0.437) (Supplementary Table S11).

## 5. Discussion

In this consecutive cohort of 192 patients with hormone receptor–positive, HER2-negative metastatic breast cancer treated with a CDK4/6 inhibitor and endocrine therapy, the baseline PNI was independently associated with both progression-free and overall survival when modelled as a continuous variable. Each one-point increase corresponded to an approximately 5% lower hazard of progression and an 8% lower hazard of death, corresponding to hazard ratios of 0.782 (95% CI 0.670–0.918) and 0.656 (95% CI 0.540–0.799) per five-point increase; because the interquartile range of the PNI in this cohort spans approximately six points (48.0–54.1), a five-point contrast describes a difference between reasonably typical patients rather than an extreme comparison. The association persisted after adjustment for age, liver metastasis and line of therapy. For progression-free survival, the estimate was also stable after additional adjustment for Ki-67 in the subgroup in whom this variable was available. For overall survival, neither the NLR nor the SIRI added prognostic information to a model containing the PNI. Serial change in the index generally added little to the baseline value.

The direction and magnitude of our findings are consistent with the largest series reported in this setting, in which a low PNI was associated with shorter progression-free and overall survival [8], and with the smaller cohort of 106 patients in whom overall survival was longer in the high-PNI group [9]. A direct numerical comparison is not possible because both studies dichotomised the index, whereas we did not. In the larger series the PNI was no longer independently associated with progression-free survival once other covariates were entered (adjusted HR 1.247, *p* = 0.170), although it remained associated with overall survival; in the present cohort the association with progression-free survival persisted in the multivariable model (HR 0.952, *p* = 0.003). Loss of statistical power is a recognised consequence of dichotomising a continuous variable [20], and the contrast between the two analyses is consistent with that explanation rather than with a difference between the cohorts themselves. The direction of the association is also concordant with reports of the PNI in other breast cancer settings, including early and node-positive disease, neoadjuvant chemotherapy and other systemic treatments for metastatic disease [13,14,21,22,23,24,25].

The threshold itself is no more stable. The median PNI in our cohort was 51.0, whereas the median in the 431-patient series was 47.5 [8]; had we adopted the same median split strategy, our threshold would have differed by more than three points in an otherwise comparable population. Conversely, applying the literature-derived value of 45 [14] to our data classified only 24 of 190 patients (12.6%) as having a low PNI, whereas the median split used in the larger series placed 52.4% of its patients in the low group [8]. These proportions are not directly comparable because the two cohorts differ, as do the thresholds, but they illustrate how differently the same label can be applied. Across breast cancer series the cut-offs applied to the same index span 40.0 to 52.8 [9,13,14,21,22,25]. A boundary that shifts in this way between similar cohorts is unlikely to reflect a biological transition, and effect estimates conditioned on it are correspondingly difficult to transfer [20]. For this reason we regard the continuous analysis as the primary evidence of the present study and the dichotomised curves as illustrative only. The width of the confidence intervals around the medians in the small low-PNI subgroup (4.4–19.6 months for progression-free survival) supports that reading.

Both the SIRI and the NLR were associated with overall survival in univariable analysis (HR 1.194, *p* = 0.001 and HR 1.034, *p* = 0.015). After adjustment for a covariate set that included the PNI, neither remained associated with outcome (*p* = 0.322 and *p* = 0.331), while the estimate for the PNI was essentially unchanged in both models. For progression-free survival neither index was significant even in univariable analysis. A prognostic association between a high NLR and worse outcome has been reported in breast cancer more broadly, including in a real-world cohort of 2218 patients treated with a CDK4/6 inhibitor [10] and in systematic reviews and meta-analyses [26,27,28]. Our findings do not contradict those reports, since the NLR was associated with overall survival in unadjusted analysis here as well; what they add is that the association did not persist once the PNI was entered into the same model. A prospective study of 776 patients that compared five nutritional assessment tools likewise found the PNI to be the most strongly associated with overall survival, although the discrimination achieved by all the indices examined was modest and a modified version of the index incorporating serum cholesterol performed better still in that cohort [29].

All three indices are derived from the same routine blood tests, and all nominally incorporate the lymphocyte count, which is thought to reflect adaptive antitumour capacity [30]. In practice, however, they measured different things: the PNI correlated with albumin and with the lymphocyte count but not with the neutrophil count, whereas the NLR and the SIRI were dominated by the neutrophil count and were closely correlated with each other. What distinguishes the PNI, therefore, is not only its formula but also the compartment it reflects, particularly the inclusion of serum albumin. Albumin is not simply a marker of dietary intake. Hypoalbuminaemia in cancer arises through several overlapping mechanisms—reduced hepatic synthesis, increased catabolism and redistribution into the extravascular compartment—which are driven in part by pro-inflammatory cytokines [31,32]. The albumin concentration may therefore reflect chronic systemic inflammation together with nutritional reserve and hepatic synthetic capacity, whereas ratios constructed from leucocyte subpopulations reflect the cellular inflammatory compartment alone. In this population, the index containing an albumin term was associated with outcome in adjusted models, whereas the purely cell-based ratios were not retained; formal comparison of the models supported this asymmetry: within this cohort neither inflammatory ratio added information to a model containing the PNI, whereas the PNI added information to models containing either ratio, and the models containing the PNI alone gave the lowest Akaike information criterion. We do not interpret this as establishing generalisable superiority of the index, which would require external validation in independent cohorts. Examining the two constituents of the index separately refined this interpretation. For overall survival the composite construction was supported: albumin and the lymphocyte count each carried information the other did not, the fixed weighting proposed by Onodera was consistent with these data, and the composite index fitted better than either component alone. For progression-free survival, by contrast, albumin accounted for the association and the lymphocyte count added nothing detectable, so that for this endpoint the index performed no better than its albumin term. The prognostic contribution of the lymphocyte count therefore appears to be specific to overall survival in this cohort. This distinction is worth stating plainly, since it qualifies the rationale for preferring a composite index over serum albumin, which is more widely measured and more readily interpreted.

This interpretation should be advanced cautiously. Albumin is also influenced by performance status, which was not consistently recorded in our cohort, and we cannot exclude that the PNI is partly acting as a surrogate for a variable we were unable to measure. The observation we can make with greater confidence is descriptive: within this study, the inflammatory ratios showed no independent association with outcome once the PNI was included, although the confidence intervals around those estimates do not exclude modest effects.

The largest series in this setting examined the baseline PNI only, and its authors identified dynamic monitoring of the index during treatment as a question for future work [8]. We were able to address it because the index was recalculated before cycles 2 and 3. The PNI remained associated with both progression-free and overall survival at each of the three time points, but the association was strongest for the pretreatment value; and once that value had been accounted for, neither the change to cycle 2 nor the change to cycle 3 was associated with either endpoint in models containing the baseline value alone. For patients who remain on treatment to the third cycle, therefore, repeating the measurement added little to the information already available before the first dose. This picture requires two qualifications. First, when the covariate set used in the primary models was added, the change to cycle 3 reached nominal significance for progression-free survival (HR 0.950, *p* = 0.028). This was one of four tests of change, was not seen for the change to cycle 2 or for overall survival, was not corrected for multiple comparisons, and was attenuated when the landmark was set at three months (*p* = 0.074) or when either liver metastasis or line of therapy was removed from the covariate set (*p* = 0.091 and *p* = 0.110), although it persisted when age was removed (*p* = 0.034). It should therefore be regarded as hypothesis-generating rather than as evidence that the index should be repeated in practice. Second, the landmark design conditions on remaining event-free and under observation until the landmark and therefore excludes patients who progressed, died or discontinued treatment earlier, so the finding applies only to patients well enough to reach that point. A change in the index would in any case be difficult to interpret causally, since improvement in albumin and lymphocyte count may follow response to treatment rather than precede it; the absence of an association avoids that difficulty here but would have complicated the interpretation of a positive result. The same pattern has been reported in a different treatment setting: in a series of 236 patients receiving neoadjuvant chemotherapy, the pretreatment PNI was associated with pathological complete response, whereas its change during treatment was not, even though changes in three other immune–nutritional indices were [33]. The neoadjuvant literature is not consistent on this point: in a smaller series of 72 patients, a smaller fall in the PNI during chemotherapy was associated with a higher rate of pathological complete response [34]. A similar pattern has been reported for body composition in the same treatment setting, in which measures obtained at three and six months were not associated with progression-free survival, whereas the baseline skeletal muscle area index remained an independent prognostic factor [35]. The pattern differed for the other two indices, which were measured at the same time points. An increase in the NLR or the SIRI by the third cycle was associated with overall survival and more strongly than any change in the PNI. These associations did not persist in sensitivity analyses. The change scores for both ratios had wide and asymmetrical distributions, and after winsorisation at the 5th and 95th percentiles neither retained any association with overall survival, with the estimate for the NLR falling essentially to the null. These apparent associations were therefore highly dependent on a small number of patients with extreme increases rather than reflecting a robust graded relationship across the cohort, and we do not regard them as evidence that serial measurement of the inflammatory ratios carries prognostic information. A rising neutrophil-to-lymphocyte ratio late in treatment would in any case be difficult to interpret causally, since it may accompany clinical deterioration, treatment-related toxicity or intercurrent infection rather than precede them. Systemic anticancer treatment is itself associated with a decline in general condition and physical function in a proportion of patients. Such deterioration would be expected to alter the neutrophil, lymphocyte and albumin compartments from which all three indices are derived. Any change observed during treatment may therefore reflect the consequences of therapy and of progressive disease as readily as it reflects an underlying prognostic state, and this ambiguity applies to the serial measurements reported here irrespective of the direction of the observed association. The contrast between the three indices is itself informative: measured in the same patients at the same time points, they behave differently enough that no general statement can be made about the value of repeated measurement. Others have reported that blood count indices change measurably within the first weeks of CDK4/6 inhibitor treatment [11]; our findings indicate that such changes are not straightforwardly prognostic.

A secondary observation concerns the classification of metastatic sites. Liver involvement was strongly associated with both endpoints (progression-free survival HR 2.414; overall survival HR 2.436), whereas lung involvement was not (*p* = 0.807 and *p* = 0.829). The composite category of visceral metastasis, defined here as involvement of the liver, lung, peritoneum, adrenal gland or brain, produced weaker estimates for both endpoints (HR 1.684 and HR 1.811). Because this composite category is widely used, including in treatment guidelines and in the previous CDK4/6 inhibitor literature [3,8,11,12], its use may dilute an association that is specific to hepatic involvement. This observation is secondary and derived from a single cohort and would need to be confirmed in independent series before it could justify a change in analytical practice.

The best response and disease control rates observed here (76.0% and 87.5%) are far higher than those reported in the largest comparable cohort, in which no patient achieved a complete response and the corresponding rates were 26.0% and 80.0% [8]. A difference of this magnitude is unlikely to reflect true differences in treatment effect and is more plausibly methodological. Part of it arises from the assessment of bone-only disease on ^18^F-FDG PET/CT in accordance with routine institutional practice, since bone lesions are not measurable under RECIST version 1.1; PET/CT permits metabolic assessment of skeletal lesions that cannot be measured anatomically, so that responses in bone-only disease were captured here but would have been classified as non-evaluable in studies restricted to measurable disease. The magnitude of this contribution is, however, limited: best response was recorded in 73.5% of patients with measurable soft-tissue disease and in 81.2% of those with bone-only disease, a difference of fewer than eight percentage points that did not reach statistical significance (*p* = 0.133). Methodological heterogeneity of assessment therefore does not appear to explain the discrepancy fully. The remainder is more plausibly attributable to the retrospective ascertainment of best response from routine clinical documentation without central radiological review, which may have contributed to differences in response classification. Differences in cohort composition, including the high proportion of patients treated in the first line (67.2%), are likely to contribute as well. This endpoint is accordingly designated an institutionally assessed best response rate and should not be compared directly with objective response rates derived from clinical trials or from series with central radiological review.

The PNI requires only serum albumin and the absolute lymphocyte count, both of which are obtained before every treatment cycle in routine practice, and it can therefore be calculated at no additional cost or delay. Other measures of nutritional status and body composition have also been associated with outcome in this setting [35]. The value of the PNI nevertheless lies in risk stratification rather than in treatment selection. Nothing in these data indicates that patients with a low PNI should receive a different regimen; in the absence of a comparator arm the index is prognostic rather than predictive. Whether the association is modifiable—that is, whether nutritional assessment and intervention before or during CDK4/6 inhibitor therapy alter outcome—cannot be addressed by an observational study and remains an open question [36,37].

The study also has several strengths. All consecutive patients treated during the study period were included, so the cohort reflects unselected real-world practice, and vital status was verified for every patient through the national death registration system. The PNI was modelled as a continuous variable, and no threshold was generated from these data, which avoids the inflation of effect estimates introduced by data-derived dichotomisation. Covariates were selected on clinical grounds rather than by univariable screening, and progression-free and overall survival were analysed with the same covariate set, allowing the two endpoints to be compared directly. The supplementary models additionally adjusted for Ki-67, the NLR and the SIRI, and the estimate for the PNI was stable across all of them.

This study has several limitations. First, it was a single-centre retrospective analysis conducted at one Turkish academic institution. Referral patterns, the local availability of individual CDK4/6 inhibitors, national reimbursement rules and institutional imaging practice all shape which patients enter such a cohort. The distribution of the PNI and of its determinants may also differ appreciably in populations with different nutritional, ethnic and socioeconomic profiles. Consecutive enrolment limits selection within the institution but cannot address selection into it, and external validity to more diverse populations therefore remains unestablished. The PNI was moreover designated the index of primary interest after the data had been assembled rather than in a prespecified analysis plan, so the findings are hypothesis-generating and require external validation.

Second, the Eastern Cooperative Oncology Group (ECOG) performance status was not consistently recorded and could not be included in the multivariable models. Because serum albumin is influenced by general condition, the observed association between the PNI and outcome may partly reflect unmeasured differences in performance status. This concern is supported by the only comparable series in which performance status was recorded, where poor performance status was considerably more frequent in the low-PNI group (16.4% versus 3.4%); in that cohort, however, the PNI remained independently associated with overall survival after adjustment for performance status [8].

Third, only 24 patients had a baseline PNI below 45, and the Kaplan–Meier estimates for that subgroup are correspondingly imprecise; the dichotomised analyses should be read as descriptive rather than as an independent test of the association.

Fourth, the pathological data have several limitations. Histological grade was available in half of the cohort and was therefore reported descriptively rather than analysed, and Ki-67 was missing in 19 patients. Pathological variables were abstracted from routine diagnostic reports without central review, and receptor status was reassessed on a metastatic specimen only where this was clinically indicated and technically feasible, most often in patients with visceral or nodal disease rather than in those with bone-only involvement. Discordance in receptor expression between primary and metastatic lesions is well described, so the tumour characteristics recorded here may not reflect the biology of the disease at the time CDK4/6 inhibitor therapy was started in every patient, and the frequency of metastatic re-biopsy was not recorded. Fifth, the cohort was heterogeneous with respect to treatment. Patients received ribociclib or palbociclib with either an aromatase inhibitor or fulvestrant across first, second and later lines of therapy; the cohort was predominantly treated with ribociclib, which limits inference about differences between individual agents. Line of therapy was adjusted for in all primary models and the PNI estimate was unchanged when the agent administered and the endocrine partner were added to each model. Nevertheless, prior systemic therapy in the metastatic setting was recorded only as line of therapy rather than as individual regimens, and residual confounding by treatment history, comorbidity burden and unmeasured patient selection cannot be excluded. Sixth, best response was determined by RECIST 1.1 in patients with measurable soft-tissue disease and by institutional ^18^F-FDG PET/CT criteria in those with bone-only disease. These methods are not equivalent, no central radiological review was performed, and the composite figure is therefore not comparable with a conventional objective response rate; response is accordingly reported separately by disease category, and the endpoint is designated an institutionally assessed best response rate. Finally, the analyses of the second and third cycle measurements are exploratory. They are conditioned on survival to a two-month landmark, they involve multiple tests that were not corrected for multiplicity, and changes in the constituent laboratory values during treatment may follow disease progression, intercurrent infection, treatment-related toxicity or a decline in general condition rather than precede them. These findings are hypothesis-generating and should not be translated into clinical monitoring practice. No external validation cohort was available.

## 6. Conclusions

In patients with hormone receptor–positive, HER2-negative metastatic breast cancer receiving a CDK4/6 inhibitor and endocrine therapy, a lower baseline PNI was associated with shorter progression-free and overall survival after adjustment for age, liver metastasis and line of therapy, when analysed as a continuous variable and without a threshold derived from the present data. The estimate was essentially unchanged when the number of involved metastatic organs, or the agent administered together with the endocrine partner, were added to each model. For overall survival, neither the NLR nor the SIRI added prognostic information to a model containing the PNI, and recalculating the index before the second and third cycles added little to the baseline value, although a change by the third cycle was associated with progression-free survival in one of four exploratory tests. For overall survival, in formal comparisons, neither inflammatory ratio added information to a model containing the PNI, whereas the PNI added information to models containing either ratio; we interpret this as incremental prognostic information within this cohort rather than as evidence of generalisable superiority. Examination of the components of the index indicated that its association with overall survival drew on both albumin and the lymphocyte count, whereas its association with progression-free survival was accounted for by albumin alone. Because all patients received the same class of therapy and no comparator arm was available, these findings support a prognostic rather than a predictive interpretation of the index; the dichotomised and serial analyses are secondary and exploratory, and the designation of the PNI as the index of primary interest followed rather than preceded assembly of the data. External validation in independent and more diverse cohorts, ideally with performance status recorded prospectively, is required before the PNI can inform clinical decision-making, and nothing in these data indicates that treatment selection should be altered on the basis of the index.

## Figures and Tables

**Table 1:** Baseline demographic, clinical and laboratory characteristics of the study cohort (*n* = 192).

Characteristic	Value	*n*
**Demographic characteristics**		
Age, years	58.1 (48.7–68.5)	192
Postmenopausal	139 (72.4)	192
Body mass index, kg/m^2^	28.0 (25.0–32.8)	191
**Comorbidity**		
Any comorbidity	132 (68.8)	192
Number of comorbidities	1 (0–3)	192
Hypertension	77 (40.1)	192
Diabetes mellitus	62 (32.3)	192
Cardiovascular disease	18 (9.4)	192
Thromboembolic event	17 (8.9)	192
Hyperlipidaemia	12 (6.3)	192
Polypharmacy (≥5 drugs)	26 (13.5)	192
**Tumour characteristics**		
Invasive breast carcinoma of no special type (NST)	145 (75.5)	192
Invasive lobular carcinoma	24 (12.5)	192
Other	23 (12.0)	192
Ki-67 index, %	20 (15–30)	173
Oestrogen receptor, % of cells	95 (90–100)	189
Progesterone receptor, % of cells	40 (1–80)	189
Progesterone receptor negative (<1%)	45 (23.8)	189
HER2 immunohistochemistry 0	158 (83.6)	189
HER2-low (IHC 1+ or 2+/ISH-negative)	31 (16.4)	189
De novo metastatic disease	105 (54.7)	192
Prior adjuvant endocrine therapy	86 (44.8)	192
**Sites of metastasis**		
Bone	164 (85.4)	192
Lymph node	84 (43.8)	192
Lung	51 (26.6)	192
Liver	48 (25.0)	192
Brain	10 (5.2)	192
Peritoneum	4 (2.1)	192
Adrenal gland	4 (2.1)	192
Visceral metastasis	98 (51.0)	192
Bone-only (±lymph node)	85 (44.3)	192
**Treatment**		
Ribociclib	143 (74.5)	192
Palbociclib	49 (25.5)	192
First line	129 (67.2)	192
Second line	35 (18.2)	192
Third line or beyond	28 (14.6)	192
Aromatase inhibitor	123 (64.1)	192
Fulvestrant	69 (35.9)	192
Dose reduction	67 (34.9)	192
**Baseline laboratory values**		
Haemoglobin, g/dL	12.0 (10.8–13.2)	192
Neutrophils,/µL	3200 (2200–4775)	192
Lymphocytes,/µL	1600 (1200–2200)	190
Monocytes,/µL	400 (300–500)	187
Platelets, ×10^9^/L	252.5 (196.3–320.3)	192
Albumin, g/L	43.0 (40.8–45.0)	190
**Inflammatory and nutritional indices**		
Prognostic nutritional index	51.0 (48.0–54.1)	190
Neutrophil-to-lymphocyte ratio	2.00 (1.31–2.97)	190
Systemic inflammation response index	0.78 (0.41–1.38)	187
**Best response**		
Complete response	14 (7.3)	192
Partial response	132 (68.8)	192
Stable disease	22 (11.5)	192
Progressive disease	24 (12.5)	192
Institutionally assessed best response rate	146 (76.0)	192
Measurable soft-tissue disease (RECIST 1.1)	72 (73.5)	98
Bone-only ± lymph node (PET/CT)	69 (81.2)	85
Other non-measurable disease	5 (55.6)	9
Disease control rate	168 (87.5)	192
**Outcomes**		
Disease progression	124 (64.6)	192
Death	83 (43.2)	192

Data are presented as median (interquartile range) for continuous variables and *n* (%) for categorical variables; percentages are based on valid observations. Ki-67 was missing in 19 patients, lymphocyte count and albumin in 2, monocyte count in 5, and body mass index in 1. Histological grade was available in 96 patients (50.0%) and is therefore not presented. IHC, immunohistochemistry; ISH, in situ hybridisation.

**Table 2:** Cox regression analyses for progression-free survival.

Variable	Univariable HR (95% CI)	*p*	Multivariable HR (95% CI)	*p*
**Baseline PNI (per 1 point)**	**0.946 (0.917–0.977)**	**<0.001**	**0.952 (0.923–0.983)**	**0.003**
**Liver metastasis**	**2.414 (1.661–3.509)**	**<0.001**	**2.114 (1.421–3.146)**	**<0.001**
**Line of therapy (per line)**	**1.239 (1.105–1.389)**	**<0.001**	**1.251 (1.106–1.416)**	**<0.001**
Age (per year)	0.996 (0.981–1.011)	0.594	0.999 (0.984–1.014)	0.917
**Visceral metastasis**	**1.684 (1.177–2.408)**	**0.004**	**—**	**—**
**Bone-only ± lymph node**	**0.650 (0.453–0.934)**	**0.020**	**—**	**—**
**Ki-67 (per 1%)**	**1.014 (1.002–1.026)**	**0.023**	**—**	**—**
SIRI (per 1 unit)	1.110 (0.998–1.235)	0.054	—	—
NLR (per 1 unit)	1.022 (0.992–1.052)	0.151	—	—
Bone metastasis	1.403 (0.804–2.448)	0.233	—	—
Dose reduction	0.875 (0.603–1.268)	0.480	—	—
Menopausal status (post vs. pre)	1.142 (0.759–1.717)	0.524	—	—
Any comorbidity	0.891 (0.608–1.307)	0.556	—	—
Body mass index (per kg/m^2^)	0.990 (0.958–1.024)	0.568	—	—
Lung metastasis	0.951 (0.636–1.423)	0.807	—	—
Ribociclib (vs. palbociclib)	1.133 (0.757–1.696)	0.543	—	—
**Fulvestrant (vs. AI)**	**1.561 (1.089–2.237)**	**0.015**	**—**	**—**
**Number of metastatic organs**	**1.632 (1.318–2.021)**	**<0.001**	**—**	**—**
De novo metastatic disease	0.957 (0.671–1.364)	0.808	—	—

HR, hazard ratio; CI, confidence interval; PNI, prognostic nutritional index; NLR, neutrophil-to-lymphocyte ratio; SIRI, systemic inflammation response index. Hazard ratios are presented to three decimal places and *p* values to three decimal places. Variables reaching *p* < 0.05 in univariable analysis are shown in bold. The multivariable model was fitted by the enter method and included baseline PNI, age, liver metastasis and line of therapy; visceral metastasis and bone-only disease were not entered into the multivariable model because they represent overlapping patterns of metastatic distribution with liver metastasis and were therefore not included simultaneously; Ki-67 was assessed in a supplementary model presented in Supplementary Table S2. The full cohort comprised 192 patients with 124 progression events. Univariable analyses were based on 192 patients (124 events) unless otherwise limited by missing data: 190 patients (122 events) for PNI and NLR, 187 (120 events) for SIRI, 191 (124 events) for body mass index, and 173 (114 events) for Ki-67. The multivariable model was fitted in the 190 patients with an evaluable baseline PNI (122 events).

**Table 3:** Cox regression analyses for overall survival.

Variable	Univariable HR (95% CI)	*p*	Multivariable HR (95% CI)	*p*
**Baseline PNI (per 1 point)**	**0.911 (0.878–0.946)**	**<0.001**	**0.919 (0.884–0.956)**	**<0.001**
**Liver metastasis**	**2.436 (1.572–3.776)**	**<0.001**	**2.060 (1.289–3.293)**	**0.003**
**Line of therapy (per line)**	**1.186 (1.019–1.381)**	**0.028**	**1.145 (0.972–1.350)**	**0.105**
Age (per year)	1.004 (0.986–1.023)	0.646	1.007 (0.988–1.026)	0.481
**SIRI (per 1 unit)**	**1.194 (1.072–1.330)**	**0.001**	**—**	**—**
**NLR (per 1 unit)**	**1.034 (1.006–1.062)**	**0.015**	**—**	**—**
**Visceral metastasis**	**1.811 (1.157–2.835)**	**0.009**	**—**	**—**
**Bone-only ± lymph node**	**0.598 (0.379–0.943)**	**0.027**	**—**	**—**
Any comorbidity	1.202 (0.738–1.957)	0.460	—	—
Body mass index (per kg/m^2^)	1.012 (0.971–1.055)	0.560	—	—
Menopausal status (post vs. pre)	1.124 (0.679–1.860)	0.649	—	—
De novo metastatic disease	1.054 (0.683–1.627)	0.811	—	—
Lung metastasis	0.948 (0.582–1.544)	0.829	—	—
Bone metastasis	1.223 (0.632–2.370)	0.550	—	—
Ribociclib (vs. palbociclib)	1.269 (0.780–2.064)	0.338	—	—
Fulvestrant (vs. AI)	1.413 (0.914–2.185)	0.120	—	—
**Number of metastatic organs**	**1.412 (1.104–1.806)**	**0.006**	**—**	**—**
Dose reduction	1.033 (0.663–1.611)	0.885	—	—
Ki-67 (per 1%)	1.000 (0.985–1.015)	0.973	—	—

Abbreviation and modelling approach as in Table 2. The full cohort comprised 192 patients with 83 deaths. Univariable analyses were based on 192 patients (83 deaths) unless otherwise limited by missing data: 190 patients (81 deaths) for PNI and NLR, 187 (79 deaths) for SIRI, 191 (83 deaths) for body mass index, and 173 (79 deaths) for Ki-67. The multivariable model was fitted in the 190 patients with an evaluable baseline PNI (81 deaths). We assessed SIRI and NLR in separate multivariable models presented in Supplementary Tables S3 and S4.

## Data Availability

The data presented in this study are available on request from the corresponding author. The data are not publicly available owing to restrictions relating to patient privacy.

## Supplementary Materials

The following supporting information can be downloaded at: https://www.mdpi.com/article/10.3390/jcm15176790/s1. Table S1: Spearman rank correlations between the three indices and their constituent laboratory values; Table S2: Multivariable Cox regression for progression-free survival with additional adjustment for Ki-67; Table S3: Multivariable Cox regression for overall survival with additional adjustment for the systemic inflammation response index; Table S4: Multivariable Cox regression for overall survival with additional adjustment for the neutrophil-to-lymphocyte ratio; Table S5: Primary multivariable models with additional adjustment for the number of involved metastatic organs; Table S6: Primary multivariable models with additional adjustment for the CDK4/6 inhibitor administered and the endocrine partner; Table S7: Serial measurements of the PNI, the NLR and the SIRI, and change from baseline, in relation to progression-free and overall survival; Table S8: Model fit statistics and likelihood ratio tests comparing the PNI with the NLR and the SIRI for overall survival; Table S9: Multivariable Cox models containing the PNI, its individual components, and both components entered separately; Table S10: Primary multivariable models excluding the patient with inoperable locoregional recurrence without documented distant metastasis; Table S11: Cycle 3 change-score models for the NLR and the SIRI before and after winsorisation; Table S12: Distribution of histological grade among evaluable cases; Table S13: Tests of the proportional hazards assumption based on scaled Schoenfeld residuals; Figure S1: Log-minus-log survival plots used to assess the proportional hazards assumption; Figure S2: Scaled Schoenfeld residuals for the baseline PNI plotted against Kaplan–Meier-transformed time.
