# Supplementary material for: Baseline and Early On-Treatment Prognostic Nutritional Index in Hormone Receptor–Positive, HER2-Negative Metastatic Breast Cancer Treated with CDK4/6 Inhibitors
Source: J Clin Med. 2026 Sep 1;15(17):6790. doi: 10.3390/jcm15176790 (PMC13566297; doi:10.3390/jcm15176790)
Supplement: Supplementary file 1 [file jcm-15-06790-s001.zip › jcm-4535510-supplementary.pdf]

Supplementary Materials

Supplementary Table S1. Spearman rank correlations between the three indices and their constituent laboratory values.

	PNI	NLR	SIRI	Albumin	Lymphocytes	Neutrophils
PNI	1.00					
NLR	−0.49	1.00				
SIRI	−0.26	0.80	1.00			
Albumin	0.74	−0.33	−0.30	1.00		
Lymphocytes	0.68	−0.45	−0.10*	0.11*	1.00	
Neutrophils	−0.04*	0.72	0.79	−0.28	0.23	1.00
Monocytes	0.15	0.24	0.72	−0.12*	0.36	0.55

PNI, Prognostic Nutritional Index; NLR, neutrophil-to-lymphocyte ratio; SIRI, systemic inflammation response index. Coefficients are Spearman rank correlations. Analyses used pairwise deletion; n = 190 for comparisons not involving the SIRI or the monocyte count and n = 187 otherwise. All coefficients were significant at P < 0.05 except those marked with an asterisk.

Supplementary Table S2. Multivariable Cox regression for progression-free survival with additional adjustment for Ki-67 (n = 171; 112 events).

Variable	HR (95% CI)	P
**Baseline PNI (per 1 point)**	**0.954 (0.923–0.986)**	**0.005**
**Liver metastasis**	**1.882 (1.225–2.889)**	**0.004**
**Line of therapy (per line)**	**1.335 (1.182–1.508)**	**<0.001**
Ki-67 (per 1%)	1.009 (0.997–1.022)	0.135
Age (per year)	1.005 (0.989–1.022)	0.510

HR, hazard ratio; CI, confidence interval; PNI, Prognostic Nutritional Index. The model was fitted by the enter method. Ki-67 was unavailable in 19 patients and was therefore not included in the primary model presented in Table 2; this supplementary model uses the same covariate set with the addition of Ki-67. It was fitted in the 171 patients with both an evaluable baseline PNI and a recorded Ki-67 index.

Supplementary Table S3. Multivariable Cox regression for overall survival with additional adjustment for the systemic inflammation response index (n = 187; 79 events).

Variable	HR (95% CI)	P
**Baseline PNI (per 1 point)**	**0.933 (0.890–0.979)**	**0.005**
**Liver metastasis**	**2.044 (1.268–3.296)**	**0.003**
Line of therapy (per line)	1.156 (0.976–1.370)	0.093
SIRI (per 1 unit)	1.076 (0.931–1.243)	0.322
Age (per year)	1.008 (0.989–1.028)	0.413

SIRI, systemic inflammation response index; other abbreviations as in Supplementary Table S1. The model was fitted by the enter method. Relative to the primary model (n = 190), three further patients were excluded owing to missing monocyte counts.

Supplementary Table S4. Multivariable Cox regression for overall survival with additional adjustment for the neutrophil-to-lymphocyte ratio (n = 190; 81 events).

Variable	HR (95% CI)	P
**Baseline PNI (per 1 point)**	**0.925 (0.887–0.965)**	**<0.001**
**Liver metastasis**	**2.073 (1.298–3.310)**	**0.002**
Line of therapy (per line)	1.155 (0.980–1.363)	0.086
NLR (per 1 unit)	1.019 (0.981–1.058)	0.331
Age (per year)	1.008 (0.989–1.027)	0.426

NLR, neutrophil-to-lymphocyte ratio; other abbreviations as in Supplementary Table S1. The model was fitted by the enter method. SIRI and NLR were modelled separately because both indices are derived from the neutrophil and lymphocyte counts and therefore convey overlapping information.

Supplementary Table S5. Primary multivariable models with additional adjustment for the number of involved metastatic organs.

Variable	HR (95% CI)	P
**Progression-free survival (n = 190; 122 events)**		
PNI (per 1-point increase)	0.956 (0.926–0.987)	0.006
Number of metastatic organs	1.293 (1.008–1.657)	0.043
Liver metastasis	1.715 (1.096–2.684)	0.018
Line of therapy	1.234 (1.091–1.395)	<0.001
Age (per year)	0.997 (0.982–1.012)	0.693
**Overall survival (n = 190; 81 deaths)**		
PNI (per 1-point increase)	0.920 (0.885–0.956)	<0.001
Number of metastatic organs	1.065 (0.792–1.433)	0.676
Liver metastasis	1.955 (1.152–3.319)	0.013
Line of therapy	1.145 (0.972–1.348)	0.105
Age (per year)	1.007 (0.988–1.026)	0.501

PNI, Prognostic Nutritional Index; HR, hazard ratio; CI, confidence interval. The number of involved organs was recorded for the whole cohort; the analysis was limited to the 190 patients with an evaluable baseline PNI. Each model was fitted by the enter method. Liver metastasis was retained alongside the organ count because hepatic involvement was the site of primary interest. The two variables carry partly the same information: the hazard ratio for liver metastasis fell from 2.114 (95% CI 1.421–3.146) in the primary progression-free survival model to 1.715 (95% CI 1.096–2.684) when the organ count was added, which is why the count was not entered into the primary models.

Supplementary Table S6. Primary multivariable models with additional adjustment for the CDK4/6 inhibitor administered and the endocrine partner.

Variable	HR (95% CI)	P
**Progression-free survival (n = 190; 122 events)**		
PNI (per 1-point increase)	0.953 (0.922–0.985)	0.005
Ribociclib (vs palbociclib)	1.099 (0.719–1.678)	0.663
Fulvestrant (vs aromatase inhibitor)	1.414 (0.972–2.056)	0.070

Liver metastasis	2.086 (1.397–3.114)	<0.001
Line of therapy	1.227 (1.077–1.400)	0.002
Age (per year)	0.997 (0.982–1.013)	0.756
**Overall survival (n = 190; 81 deaths)**		
PNI (per 1-point increase)	0.916 (0.880–0.955)	<0.001
Ribociclib (vs palbociclib)	1.227 (0.731–2.059)	0.439
Fulvestrant (vs aromatase inhibitor)	1.398 (0.886–2.207)	0.150
Liver metastasis	2.029 (1.263–3.260)	0.003
Line of therapy	1.126 (0.945–1.341)	0.184
Age (per year)	1.007 (0.987–1.027)	0.484

PNI, Prognostic Nutritional Index; HR, hazard ratio; CI, confidence interval. Ribociclib was administered to 143 patients (74.5%) and palbociclib to 49 (25.5%); the endocrine partner was an aromatase inhibitor in 123 patients (64.1%) and fulvestrant in 69 (35.9%). Each model was fitted by the enter method. Fulvestrant was associated with progression-free survival in univariable analysis (HR 1.561, 95% CI 1.089–2.237, P = 0.015) but not after adjustment, which is consistent with its preferential use in later lines; the coefficients for the treatment variables should therefore not be interpreted as treatment effects.

Supplementary Table S7. Serial measurements of the PNI, the NLR and the SIRI, and change from baseline, in relation to progression-free and overall survival (landmark analysis from two months).

Distribution of the index and of its change from baseline (n = 183)

	**Median (IQR)**	**Range**
PNI at baseline	51.0 (48.0–54.0)	26.5 to 67.5
PNI before cycle 2	49.5 (46.0–53.5)	24.5 to 64.0
PNI before cycle 3	49.5 (46.5–53.0)	25.0 to 68.0
Change to cycle 2	−1.5 (−4.5 to 1.5)	−13.5 to 12.5
Change to cycle 3	−1.5 (−4.0 to 1.5)	−19.5 to 10.0

Hazard ratios with the baseline value as the only other term

**Variable**	**HR (95% CI)**	**P**
**Progression-free survival (n = 181; 115 events)**		
PNI at baseline	0.954 (0.923–0.986)	0.005
PNI before cycle 2	0.963 (0.935–0.993)	0.015
PNI before cycle 3	0.961 (0.931–0.991)	0.011
Change to cycle 2, adjusted for baseline	0.986 (0.946–1.029)	0.527
Change to cycle 3, adjusted for baseline	0.982 (0.940–1.025)	0.399
**Overall survival (n = 183; 77 deaths)**		
PNI at baseline	0.917 (0.882–0.954)	<0.001
PNI before cycle 2	0.935 (0.903–0.968)	<0.001
PNI before cycle 3	0.938 (0.903–0.974)	<0.001
Change to cycle 2, adjusted for baseline	0.973 (0.926–1.023)	0.284
Change to cycle 3, adjusted for baseline	0.984 (0.934–1.037)	0.558

The same models with the covariate set used in the primary analyses added (age, liver metastasis and line of therapy)

Variable	HR (95% CI)	P
**Progression-free survival (n = 181; 115 events)**		
PNI at baseline	0.959 (0.928–0.991)	0.013
PNI before cycle 2	0.962 (0.933–0.991)	0.011
PNI before cycle 3	0.949 (0.921–0.979)	<0.001
Change to cycle 2, adjusted for baseline	0.977 (0.935–1.022)	0.311
Change to cycle 3, adjusted for baseline	0.950 (0.907–0.994)	0.028
**Overall survival (n = 183; 77 deaths)**		
PNI at baseline	0.925 (0.889–0.963)	<0.001
PNI before cycle 2	0.934 (0.902–0.967)	<0.001
PNI before cycle 3	0.936 (0.902–0.972)	<0.001
Change to cycle 2, adjusted for baseline	0.961 (0.913–1.013)	0.138
Change to cycle 3, adjusted for baseline	0.969 (0.918–1.022)	0.250

Change in the NLR and the SIRI, adjusted for the corresponding baseline value and for age, liver metastasis and line of therapy

Variable	HR (95% CI)	P
**Progression-free survival (n = 181; 115 events)**		
NLR, change to cycle 2	1.029 (0.980–1.081)	0.249
NLR, change to cycle 3	1.062 (0.975–1.156)	0.169
SIRI, change to cycle 2	0.964 (0.707–1.314)	0.815
SIRI, change to cycle 3	1.307 (0.973–1.754)	0.075
**Overall survival (n = 183; 77 deaths)**		
NLR, change to cycle 2	0.995 (0.937–1.057)	0.870
NLR, change to cycle 3	1.134 (1.038–1.239)	0.005
SIRI, change to cycle 2	0.864 (0.593–1.259)	0.447
SIRI, change to cycle 3	1.443 (1.086–1.919)	0.012

PNI, Prognostic Nutritional Index; NLR, neutrophil-to-lymphocyte ratio; SIRI, systemic inflammation response index; HR, hazard ratio; CI, confidence interval. All change-score models were restricted to the two-month landmark cohort and contained the corresponding baseline value; the number of patients contributing to each model varies slightly with the availability of the individual measurements. Hazard ratios are per one-point increase. Analyses were restricted to the 183 patients in whom a complete blood count and serum albumin were available at all three time points, and survival time was measured from a landmark at two months; patients with an event before the landmark were excluded. Models containing a change term also contained the corresponding baseline value, and the hazard ratio shown for change is that remaining after adjustment for baseline. Each model was fitted by the enter method. The upper panel of hazard ratios shows models containing the change term and the baseline value only; the lower panel repeats these models with the covariate set used in the primary analyses. The final panel shows the corresponding models for the NLR and the SIRI.

Supplementary Table S8. Model fit statistics and likelihood ratio tests comparing the PNI with the NLR and the SIRI for overall survival.

Model	−2 log likelihood	AIC
Covariates + PNI (n = 190)	733.139	741.14
Covariates + NLR (n = 190)	744.657	752.66
Covariates + PNI + NLR (n = 190)	732.378	742.38
Covariates + PNI (n = 187)	714.702	722.70
Covariates + SIRI (n = 187)	721.937	729.94
Covariates + PNI + SIRI (n = 187)	713.773	723.77

Comparison	Likelihood ratio χ^2 (df = 1)	P
NLR added to a model containing the PNI	0.761	0.383
PNI added to a model containing the NLR	12.279	<0.001
SIRI added to a model containing the PNI	0.929	0.335
PNI added to a model containing the SIRI	8.164	0.004

PNI, Prognostic Nutritional Index; NLR, neutrophil-to-lymphocyte ratio; SIRI, systemic inflammation response index; AIC, Akaike information criterion. Covariates were age, liver metastasis and line of therapy. All models within a comparison were fitted by the enter method on identical observations: comparisons involving the NLR in the 190 patients with an evaluable baseline PNI, and those involving the SIRI in the 187 patients with an evaluable monocyte count.

Supplementary Table S9. Multivariable Cox models containing the PNI, its individual components, and both components entered separately (n = 190).

Progression-free survival (122 events)

Model	Component HR (95% CI); P	−2LL / AIC
PNI (per 1 point)	0.952 (0.923–0.983); P = 0.003	1100.842 / 1108.84
Albumin alone (per 1 g/L)	0.936 (0.900–0.974); P = 0.001	1100.592 / 1108.59
Lymphocytes alone (per 200/µL)	0.967 (0.918–1.018); P = 0.204	1107.980 / 1115.98
Both components	albumin 0.937 (0.900–0.976), P = 0.002; lymphocytes 0.975 (0.927–1.025), P = 0.324	1099.597 / 1109.60

Overall survival (81 deaths)

Model	Component HR (95% CI); P	−2LL / AIC
PNI (per 1 point)	0.919 (0.884–0.956); P < 0.001	733.139 / 741.14
Albumin alone (per 1 g/L)	0.921 (0.880–0.965); P < 0.001	739.580 / 747.58
Lymphocytes alone (per 200/µL)	0.909 (0.845–0.978); P = 0.011	742.630 / 750.63
Both components	albumin 0.921 (0.877–0.966), P < 0.001; lymphocytes 0.916 (0.854–0.982), P = 0.014	733.125 / 743.12

Likelihood ratio tests (1 df)

Comparison	Progression-free survival	Overall survival
PNI versus both components with free coefficients	χ^2 = 1.245; P = 0.265	χ^2 = 0.014; P = 0.906
Lymphocyte count added to albumin	χ^2 = 0.995; P = 0.319	χ^2 = 6.455; P = 0.011
Albumin added to lymphocyte count	χ^2 = 8.383; P = 0.004	χ^2 = 9.505; P = 0.002

PNI, Prognostic Nutritional Index; AIC, Akaike information criterion. All models were fitted by the enter method in the 190 patients with an evaluable baseline PNI and additionally contained age, liver metastasis and line of therapy. Expressed in the units recorded here the index equals albumin (g/L) plus 0.005 × the lymphocyte count (/µL), so that one index point corresponds to 1 g/L of albumin or 200 lymphocytes/µL; the model containing the PNI is therefore nested within the model containing both components with freely estimated coefficients.

Supplementary Table S10. Primary multivariable models excluding the patient with inoperable locoregional recurrence without documented distant metastasis (n = 189).

Variable	Progression-free survival, HR (95% CI)	Overall survival, HR (95% CI)
Baseline PNI (per 1 point)	0.953 (0.923–0.983); P = 0.003	0.919 (0.884–0.956); P < 0.001
Liver metastasis	2.129 (1.430–3.171); P < 0.001	2.039 (1.276–3.259); P = 0.003
Line of therapy (per line)	1.252 (1.106–1.416); P < 0.001	1.143 (0.969–1.347); P = 0.112
Age (per year)	0.999 (0.984–1.015); P = 0.925	1.007 (0.988–1.026); P = 0.483

HR, hazard ratio; CI, confidence interval; PNI, Prognostic Nutritional Index. Models were fitted by the enter method. Estimates were essentially identical to those obtained in the full cohort of 190 patients with an evaluable baseline PNI (Tables 2 and 3).

Supplementary Table S11. Cycle 3 change-score models for the NLR and the SIRI before and after winsorisation.

Change score	HR (95% CI)	P
ΔNLR to cycle 3, unmodified	1.134 (1.038–1.239)	0.005
ΔNLR to cycle 3, winsorised at the 5th and 95th percentiles	0.989 (0.839–1.166)	0.897
ΔSIRI to cycle 3, unmodified	1.443 (1.086–1.919)	0.012
ΔSIRI to cycle 3, winsorised at the 5th and 95th percentiles	1.111 (0.853–1.446)	0.437

NLR, neutrophil-to-lymphocyte ratio; SIRI, systemic inflammation response index; HR, hazard ratio; CI, confidence interval. All models were fitted by the enter method in the two-month landmark cohort and contained the corresponding baseline value together with age, liver metastasis and line of therapy. Winsorisation limits were −3.52 and 1.93 for the change in the NLR and −2.71 and 0.96 for the change in the SIRI, and were specified before the models were refitted. Neither association persisted once extreme change scores were constrained.

Supplementary Table S12. Distribution of histological grade among evaluable cases.

Histological grade	n	% of evaluable cases
Grade 1	5	5.2
Grade 2	61	63.5
Grade 3	30	31.2
Total evaluable	96	100.0

Histological grade was recorded in 96 of 192 patients (50.0%) and was therefore reported descriptively only and not entered into any regression model. Percentages are calculated among evaluable cases.

Supplementary Table S13. Tests of the proportional hazards assumption based on scaled Schoenfeld residuals.

Outcome and covariate	χ^2 (df = 1)	P
Progression-free survival		
Baseline PNI (per 1 point)	0.157	0.692
Age (per year)	0.341	0.559
Liver metastasis	2.142	0.143

Line of therapy (per line)	0.215	0.643
GLOBAL (df = 4)	2.432	0.657
Overall survival		
Baseline PNI (per 1 point)	0.941	0.332
Age (per year)	2.929	0.087
Liver metastasis	0.462	0.497
Line of therapy (per line)	2.620	0.106
GLOBAL (df = 4)	6.471	0.167

PNI, Prognostic Nutritional Index. Scaled Schoenfeld residuals were computed for each covariate of the primary multivariable models, fitted by the enter method with the Breslow approximation for tied event times in the 190 patients with an evaluable baseline PNI (122 progression events; 81 deaths). Residuals were correlated with Kaplan–Meier-transformed time. No individual or global test reached statistical significance, which indicates an absence of statistically significant evidence of violation rather than confirmation that the assumption holds.

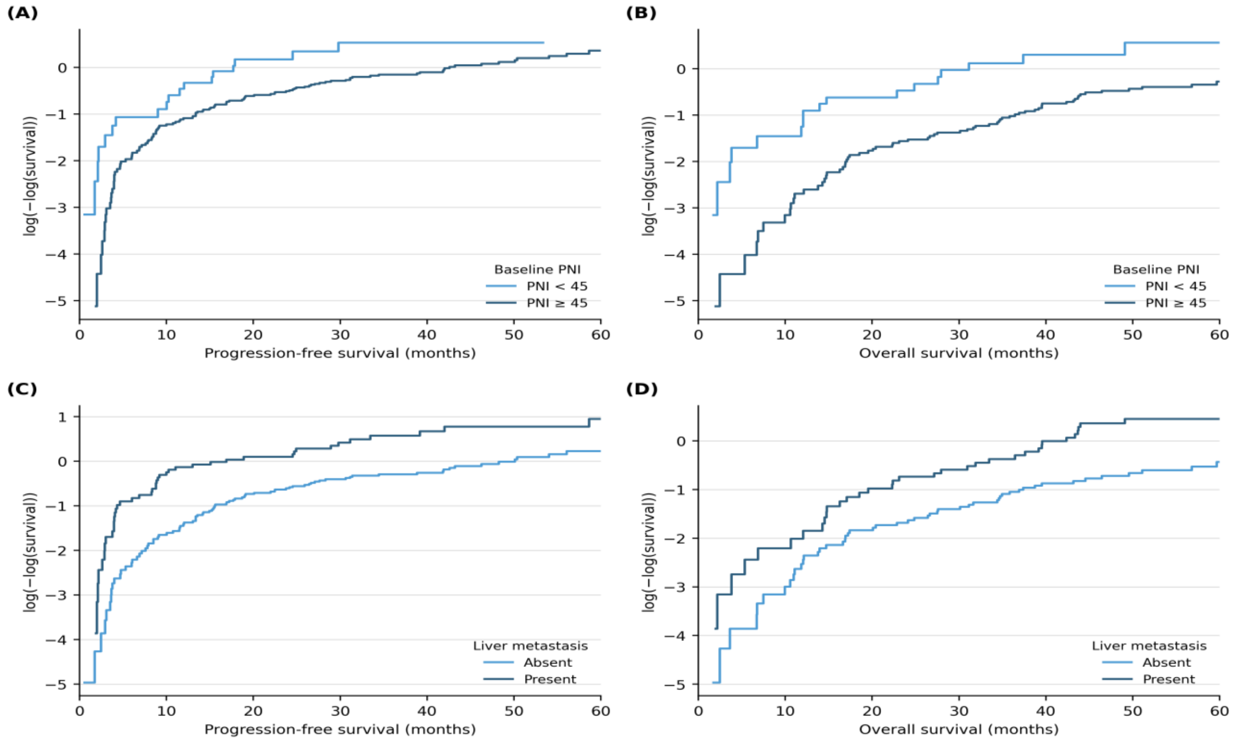

Supplementary Figure S1. Log-minus-log survival plots used to assess the proportional hazards assumption. (A) Progression-free survival by baseline Prognostic Nutritional Index (PNI < 45 versus PNI ≥ 45); (B) overall survival by baseline PNI; (C) progression-free survival by liver metastasis; (D) overall survival by liver metastasis. In each panel the curves for the two groups are approximately parallel and do not cross over the follow-up period, consistent with proportional hazards. Curves were plotted from Kaplan–Meier estimates; the vertical axis is log(−log(survival)) and the horizontal axis is time in months, truncated at 60 months.

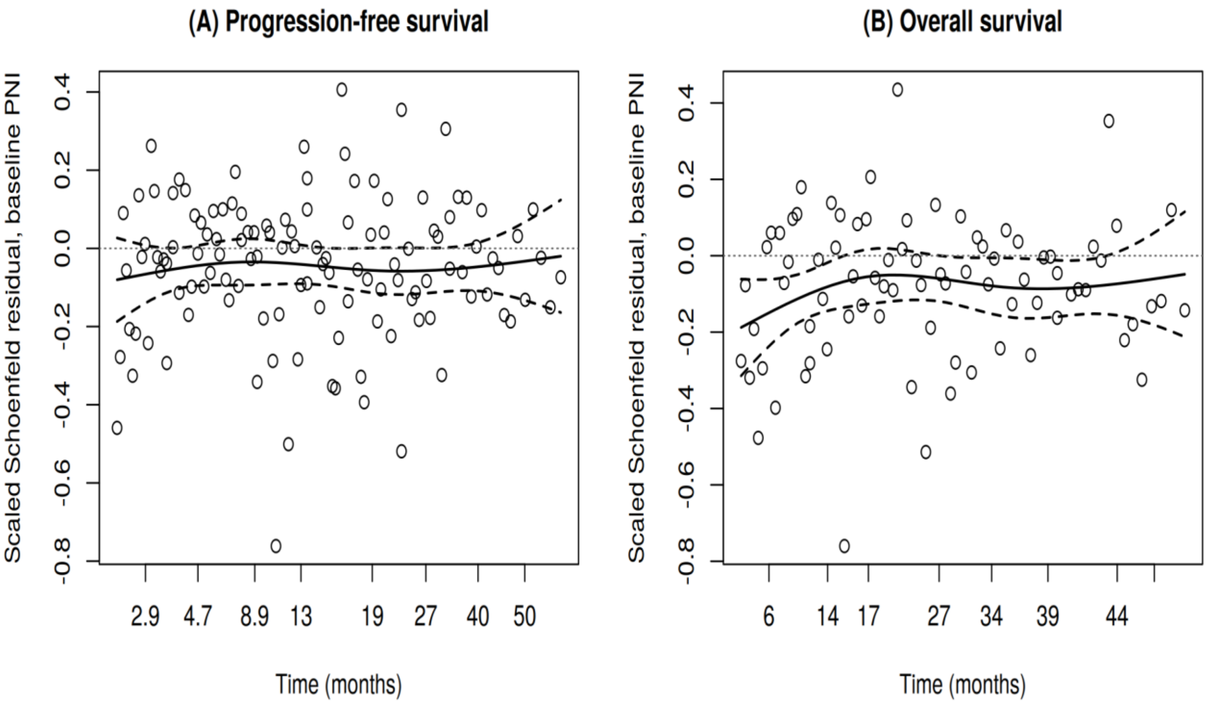

Supplementary Figure S2. Scaled Schoenfeld residuals for the baseline PNI plotted against Kaplan–Meier-transformed time. (A) Progression-free survival; (B) overall survival. The solid line is a smoothed fit with dashed pointwise confidence bands, and the dotted horizontal line marks a residual of zero. Neither panel shows a systematic trend with time, consistent with the formal tests reported in Supplementary Table S13.
